# Application of variation theory in STEM education: A comprehensive guideline for STEM teachers

**DOI:** 10.1016/j.mex.2023.102500

**Published:** 2023-11-27

**Authors:** Mahbub Hasan, Md Shahadat Hossain Khan, A.K.M. Foysal Ahmed

**Affiliations:** a Islamic University of Technology, Gazipur, Bangladesh; b International University of Business Agriculture and Technology, Dhaka, Bangladesh

**Keywords:** Variation theory, STEM, Engineering education, Lesson plan, Teaching method, Patterns of variation, Structure of awareness, Variation Theory in STEM Teaching (VTST)

## Abstract

Incorporating appropriate pedagogical strategies has been instrumental in the development of a favorable learning environment for holistic learning, particularly in Science, Technology, Engineering, and Mathematics (STEM) education. However, there is growing evidence of the need to advance the existing methods, through more robust and comprehensive fieldwork grounded in design study. It has been shown that the lack of guidance in lesson planning using variation theory is also thought to require particular attention, as the accuracy of the student learning relies significantly on the planning of the lesson. However, little research has outlined the strategy to be followed for incorporating variation theory. This article proposes a method of preparing lesson plans to systematically implement the variation theory in STEM classrooms. The method seeks to design and implement current techniques as follows:

• According to variation theory, teachers must create the necessary conditions for learning for the students to recognize what they are expected to learn.
• To create the necessary conditions for learning, teachers define the key aspects of the topic to be covered and use different patterns of variations in teaching those contents, such as contrast, separation, generalization, and fusion.
• Finally, teachers focus on the key aspects one by one or simultaneously to grab students’ attention.

## Graphical abstract

**Figure:**
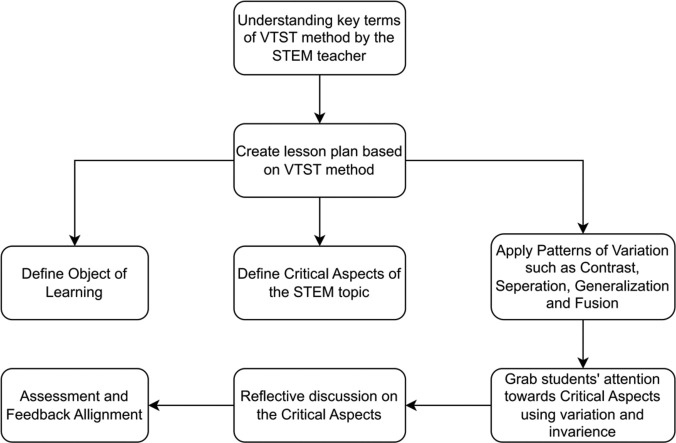

Specifications table

**Table:** 

Subject area:	Engineering
More specific subject area:	Engineering Education
Name of your method:	Variation Theory in STEM Teaching (VTST)
Name and reference of original method:	F. Marton and M. F. Pang, “On some necessary conditions of learning,” The Journal of the Learning sciences, vol. 15, no. 2, pp. 193–220, 2006.
Resource availability:	https://www.tandfonline.com/doi/abs/10.1207/s15327809jls1502_2

## Method details

### Introduction

Teaching methods strongly impact student learning [1,2]. The effectiveness of teaching methods, especially in promoting successful learning, is vital [3]. Over time, several educational methods have arisen, each having pros and cons. Effective instructional practices highlight how students acquire and assimilate knowledge, impacting academic performance [1,4]. In this context, the variation theory illuminates the need to create learning environments to help students achieve their goals [5]. In order to effectively address the multifaceted learning requirements of their students, educators must adapt their teaching methods to the continuously changing educational scene [6]. Marton and Pang's Variation Theory provides a powerful framework that focuses on purposeful variations in learning circumstances to encourage in-depth understanding among learners [5].

Variation theory has been practiced in several educational research disciplines such as clinical education [7] and language learning [8], and students benefit from this theory by increasing their understanding of the relevant field. When STEM (Science, Technology, Engineering, and Mathematics) education is the focus, variation theory becomes increasingly important because STEM courses' intricate interplay of abstract concepts and real-world applications requires a unique educational strategy that goes beyond rote memorizing to foster conceptual understanding [9], [10], [11]. With its focus on systematically adjusting essential elements to improve conceptual understanding, variation theory is ideal for complex STEM topics [12]. This alignment allows variation theory to unlock STEM education's potential by helping students connect with difficult ideas and apply them in real-life situations.

The application of variation theory in STEM education, as compared to other academic disciplines, necessitates careful deliberation due to the unique characteristics of STEM content. Within the STEM disciplines, specifically in domains such as physics, chemistry, biology, mathematics, and engineering, where learning revolves around abstract principles and concepts, variation theory plays a vital role by elucidating the interconnections among the critical aspects and cultivating a deep understanding of concepts [13]. Additionally, in STEM, particularly in the filed of engineering education there has been a specific focus on outcome-based education where students are provided with necessary knowledge and skills to prepare them for the professional work [14]. In contrast, applying variation theory to disciplines apart from STEM, such as history, literature or religious education, may direct attention toward divergences in historical contexts, interpretations, or perspectives [15]. Although variation theory's fundamental principles apply to all fields of study, the particular characteristics of variations and the prioritized critical elements can differ considerably. Nevertheless, this article is focused on examining the potential contribution of variation theory in STEM education, emphasizing its capacity to illuminate complex scientific connections through effective lesson planning.

Despite the urgent need to include variation theory in STEM education, there are limited articles that outline the implementation process of variation theory. The theoretical basis and potential benefits of variation theory in STEM education are recognized [16], but there are no systematic, step-by-step guidelines available for STEM educators, which would allow them to replicate the method. Without practical guidance, variation theory cannot be seamlessly integrated into educators' pedagogical approaches. This research fills this gap by providing a variation theory-based paradigm for STEM lesson planning. This study increases STEM pedagogy dialogue by bridging this gap and applying variation theory in the classroom.

## Key components of variation theory

**Fig. 1:**
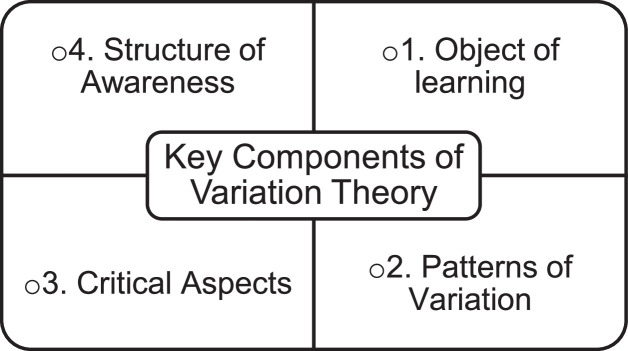
Key components of Variation Theory.

In order to apply variation theory in teaching STEM content, the first attempt that STEM teachers need to make an effort is to understand the key components of the variation theory. Unless they acquire a clear concept of the key terms concerning variation theory, teachers may struggle to adapt it in their teaching. In fact, the first step of good teaching is to plan the lesson in such a way that helps students achieve the learning objectives [17,18]. To develop a comprehensive lesson plan incorporating variation theory, the understanding of the key components is the prerequisite, without which teachers may end up planning a lesson, in which they pretend to be following variation theory, but in practice, it is not. The key components needed for the teachers to develop a lesson plan are presented in Fig. 1, which are: (i) object of learning, (ii) patterns of variation, (iii) critical aspects, and (iv) structure of awareness. A detailed explanation of each component along with their implications is discussed in the following sections.

## Object of learning

Pang and Ki defined the object of learning as - “the development of a certain powerful way of experiencing the phenomenon in question and, ultimately, is based on the learner's capability to handle novel situations in which that phenomenon occurs” [19]. In his book, “Necessary Conditions of Learning”, Marton mentioned, “for every object of learning, and for every learner, there are critical aspects and critical features, which the learners have to become able to discern” [20]. Thoughtful development of the object of learning is needed as a prerequisite for a thriving teaching-learning environment, in terms of the intended learning outcome.

**Fig. 2:**
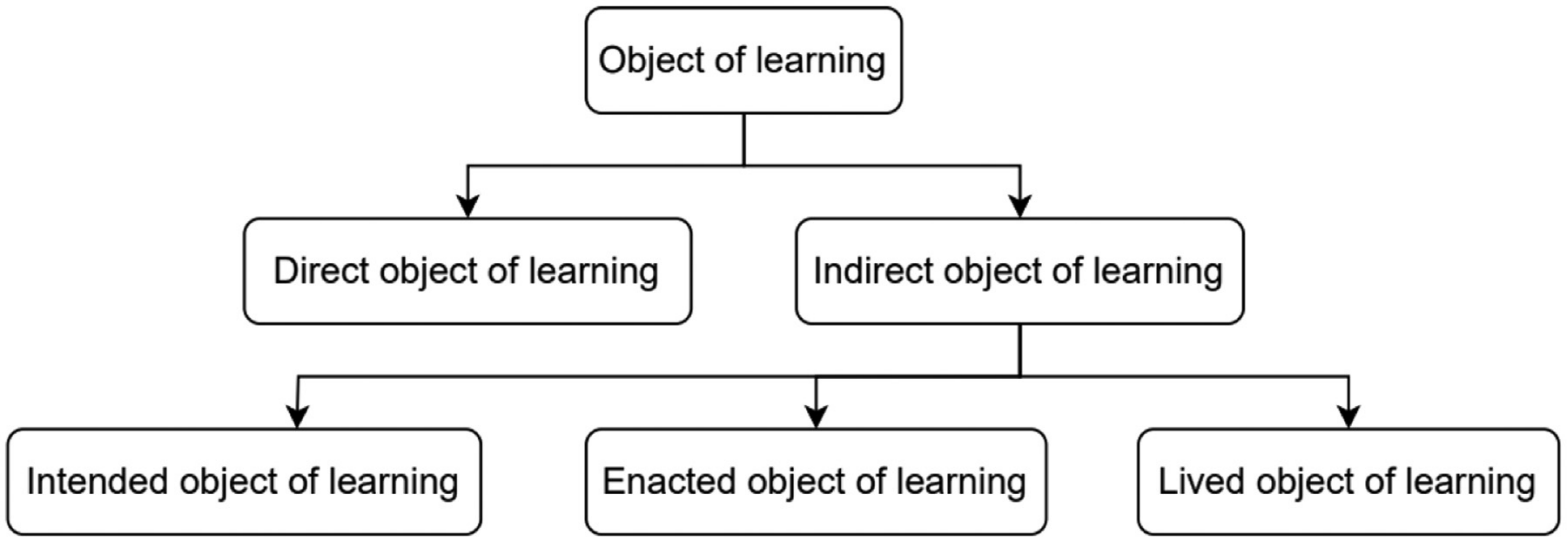
Object of learning.

As shown in Fig. 2, the object of learning has two aspects: a) direct object of learning and b) indirect object of learning [5]. The direct object of learning refers to the contents that need to be covered as a part curriculum. Whereas the indirect object of learning refers to the capability of using that content in an effective way by implementing the variation theory. This also focuses on discernment where students pay attention to what they are trying to learn. In other words, this indirect object of learning may also be referred to as the necessary conditions of learning [20].

The indirect object of learning becomes clearer when we analyze how it appears to particular people (for example, teacher, observer, student). Marton and Pang mentioned three possible ways the indirect object of learning may be experienced: 1) intended object of learning 2) enacted object of learning and 3) lived object of learning [5]. When the capability of the finalized design (objects of learning) that are seen by the course teacher, in terms of the discernment of the students, are referred to ‘intended object of learning’ [21]. A lesson plan is an example of an intended object of learning. When an observer can see (from his perspective), what is possible for these students to learn, is referred to as ‘enacted object of learning’. The objects of learning that come across the discernment of the student are referred to as ‘lived object of learning’, which can go beyond the plan and the teacher's enacted object of learning. How students perceive the object of learning, shapes their learning outcome. Different people perceive the same situation in different ways because the designed pattern (object of learning) may not be experienced by all learners in the same way. This is the point of departure where the variation theory originated from.

It is important to understand the difference between the object of learning and the learning objective when implementing variation theory in classrooms. Object of learning deals with the content to be covered, whereas learning objective focuses on the extent to which the student will be able to comprehend the concepts related to the content discussed in the classroom. Learning objectives are concise statements that explain what teachers anticipate their students will be able to do or exhibit as a direct result of the instruction they receive. They are often more specific, measurable, and action-oriented, which provides clear criteria for evaluating a student's knowledge of the topic being taught. Teachers may sometimes consider Bloom's Taxonomy as a reference to develop the learning objectives, which may sometimes refer to instructional objectives too [22]. The Object of Learning is the starting point from which Learning Objectives are developed; these objectives serve to direct instruction toward the desired level of understanding.

## Patterns of variation

**Fig. 3:**
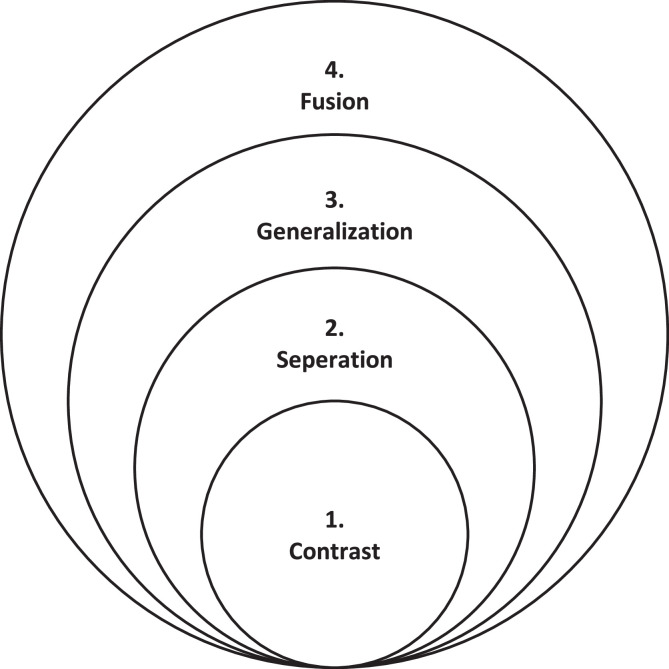
Patterns of variation.

Before implementing variation theory in the classroom, instructors must be familiar with the four primary patterns of variation proposed by variation theory: Contrast, Separation, Generalization, and Fusion [23] (see Fig. 3). The four patterns are mutually inclusive, and their complexity increases as it moves from contrast to Fusion, which is presented diagrammatically in Fig. 3. These patterns, which are founded on the notions of variation theory, aim to foster profound conceptual comprehension among learners by introducing variations and invariance on purpose into learning environments. The concept of variation and invariance refers to the discussion of an aspect of a topic (variation), while other aspects of the same topic remain constant (invariant), meaning there will be no discussion on the other aspects. The application of these patterns enables teachers to improve teaching methods that are suited to various topic areas, thus increasing the overall efficacy of learning experiences. The four patterns of variation are described in the following section.

### Contrast pattern: Illuminating essential distinctions

The Contrast pattern entails giving examples that differ in one critical aspect while remaining in all other factors constant. Teachers can direct students' attention to the varying attributes by skilfully manipulating these variations, enabling them to identify and grasp essential distinctions within a specific concept or reality. Understanding this pattern enables educators to create learning scenarios that highlight key differences and increase students' understanding of fundamental concepts.

Example: The educator demonstrates the concept of “volume” to middle school students using two cuboids with similar lengths and widths. Nevertheless, one cuboid is taller than the other. Students can determine that the volume grows proportionally with height by altering the height while holding the other dimensions constant.

### Separation pattern: Separating interrelated impacts

Through the Separation pattern, educators provide examples that vary in numerous critical aspects concurrently. This strategy enables learners to discern the various effects of each variable on the overall notion, fostering their capacity to recognize underlying linkages and dependencies. By using the Separation pattern, teachers can encourage a more thorough comprehension of complicated subjects and enable students to draw meaningful connections between distinct components.

Example: Using two different plant species exposed to various degrees of sunlight, carbon dioxide, and water availability, the instructor of high school biology students explains the concept of “photosynthesis” to the class. Students can examine and evaluate the various influences of light, CO2, and water on the rate of photosynthesis by simultaneously adjusting these elements.

### Generalization pattern: Identifying universal principles

The purpose of the Generalization design pattern is to reveal underlying ideas or concepts that present themselves in various contexts. Educators teach students to identify universal laws and patterns relevant to a variety of circumstances by offering similar examples with shared basic traits. Familiarity with the Generalization pattern enables teachers to enhance students' capacity to generalize underlying concepts from a variety of situations, resulting in more robust and transferrable learning results.

Example: An illustration of this is when a math teacher demonstrates “geometric transformations” by using various shapes (such as triangles and rectangles) and the pictures they take after being translated, rotated, and reflected. Students can generalize that all translations preserve lengths, all rotations preserve angles, and all reflections produce mirror images by finding the common characteristics of each transformation.

### Fusion pattern: Emphasizing holistic understanding

The Fusion pattern involves the strategic combination of numerous interconnected concepts or elements into a single instance. By contextualizing interconnection, educators improve students' knowledge of how various pieces work together to form a coherent understanding. Using the Fusion pattern permits educators to design learning experiences that combine multiple concepts, encouraging students to comprehend the holistic structure of complex topics and fostering higher-order thinking skills.

Example: In a first-year engineering class, students measure current (I) and voltage (V) across a resistor using a battery, ammeter, and voltmeter in a classroom. The teacher starts with a standard battery voltage and resistor resistance, and students measure the current. The teacher then adds a variation by raising resistance while maintaining battery voltage. Students see a decline in current and realize resistance inversely affects current. Another variation shifts the battery's voltage while maintaining a constant resistance, helping students understand voltage and current. Through fusion pattern, students learn how changes in one parameter impact the others, completing their understanding of voltage, current, and resistance in electric circuits.

In order to enhance student learning experiences, educators must have a comprehensive understanding of Variation Theory and its core patterns of variation. Teachers can improve the quality and efficacy of classroom learning by implementing the Contrast, Separation, Generalization, and Fusion patterns into their instructional design. This journal article emphasizes the significance of educators being familiar with these patterns and provides practical insights and pedagogical consequences for implementing them. With this information, educators may construct dynamic and engaging learning environments that encourage deeper conceptual understanding among students, empowering them to excel in a variety of academic and practical endeavors.

## Critical aspects

According to variation theory, a critical aspect is a feature or dimension of the object of learning that is changed on purpose in learning situations. These are the key elements that students need to recognize and comprehend in order to get a deep and meaningful understanding of the subject [19]. By figuring out what is important, students can identify patterns, find general principles, and make connections. This leads to a deeper understanding of concepts and better knowledge transfer.

The role of the teacher is vital in determining the critical aspects of a topic of discussion. The selection of critical aspects depends on the unique learning objectives, the topic's complexity, and the learners' proficiency level. Educators can construct interesting and effective learning situations based on the critical aspects that respond to students' different learning requirements and promote successful knowledge acquisition by skillfully designing variations and invariance around the object of learning. When identifying the critical aspects of a STEM topic, teachers need to consider the important features that students need to experience to get a holistic understanding of the topic. An example from the science topic is presented that may be useful for the STEM teachers to identify the critical aspects for their science topic.

**Fig. 4:**
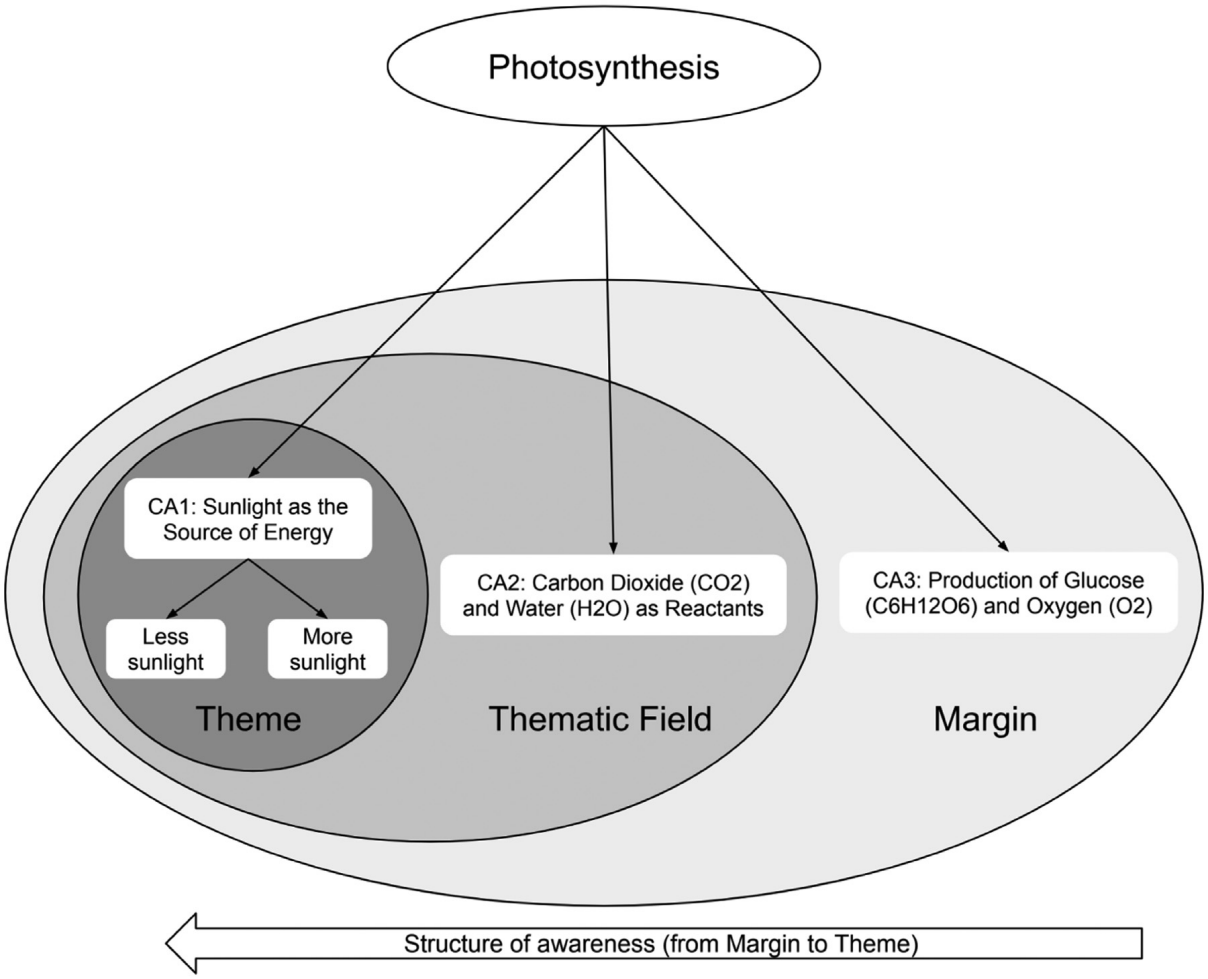
Development of Critical Aspects according to VTST method. Adapted from [24].

Example: In explaining the concept of “photosynthesis,” one of the critical aspects might be the “role of sunshine as the Source of Energy”. By exhibiting variants of different plants exposed to varying degrees of sunshine while keeping other factors constant, students can discover the significance of sunlight as a vital aspect in the photosynthetic process. Other critical aspects may be Carbon Dioxide (CO2) and Water (H2O) as reactants, and the Production of Glucose (C6H12O6) and Oxygen (O2). Fig. 4 presents a diagrammatic view of the photosynthesis process along with its critical aspects.

### Critical aspect 1 (CA1): Need of Sunlight

One of the most important features to understand the photosynthesis process is to understand the impact of light on the process. Depending on the intensity of sunlight, the rate of the photosynthesis process varies, which further produces glucose and oxygen from carbon dioxide and water. Students need to discern this critical aspect in order to understand the photosynthesis process.

### Critical aspect 2 (CA2): Need of (CO2) and (H2O)

Another important feature of the photosynthesis process is to understand the need for carbon dioxide (CO2) and water (H2O). Students should understand that plants collect carbon dioxide from the air through pores called stomata and water from the soil through their roots. In the presence of sunshine and chlorophyll, these reactants conduct a sequence of chemical processes to produce glucose and oxygen.

### Critical aspect 3 (CA3): Production of C6H12O6 and O2

Glucose (C6H12O6) and oxygen (O2) are the final products of the photosynthesis process. Students must comprehend that glucose is a sort of stored energy that plants employ for growth and sustenance, whereas oxygen is generated as a byproduct, sustaining the lives of other species, including animals.

Merely identifying the critical aspects may not guarantee that students will discern them all unless they are placed in students’ structure of awareness. Carefully altering the critical aspects to the areas of Theme, Thematic Field, and Margin may ensure that students' lived object of learning will be achieved. Therefore, understanding how students’ structure of awareness is utilized is crucial to creating the necessary conditions for learning. An explanation of the use of the structure of awareness is described in the following section.

## Structure of awareness

The structure of awareness refers to how students become aware of the critical aspects and variations provided in the learning setting [24]. It involves the cognitive processes of the students as well as their capacity to identify, distinguish, and determine the key characteristics within the variations. As the awareness of the critical aspects develops, students can draw out deeper connections and meanings from the variances, enabling a more comprehensive and holistic understanding of the subject. By carefully choosing the critical aspects that are essential to achieving the learning objectives, teachers can intentionally build learning situations that have an impact on the students’ structure of awareness. Through this process, teachers can help students become more focused in their awareness of a specific critical aspect along with its dimensions of variation.

Refer to the previous example of the STEM topic in Fig. 4, to help students understand photosynthesis, instructors need to focus on each critical aspect one by one to evolve students' structure of awareness, so students begin to discern the patterns and invariances connected to sunlight's effect on photosynthesis. Students may observe that plants exposed to more sunshine produce more glucose or oxygen than plants exposed to less sunlight (see Fig. 4. – Theme). Learners get a deeper grasp of photosynthesis as a result of this increased awareness of the connection between sunlight and the process, and they come to comprehend the crucial function that sunlight plays in promoting plant development and energy generation.

**Fig. 5:**
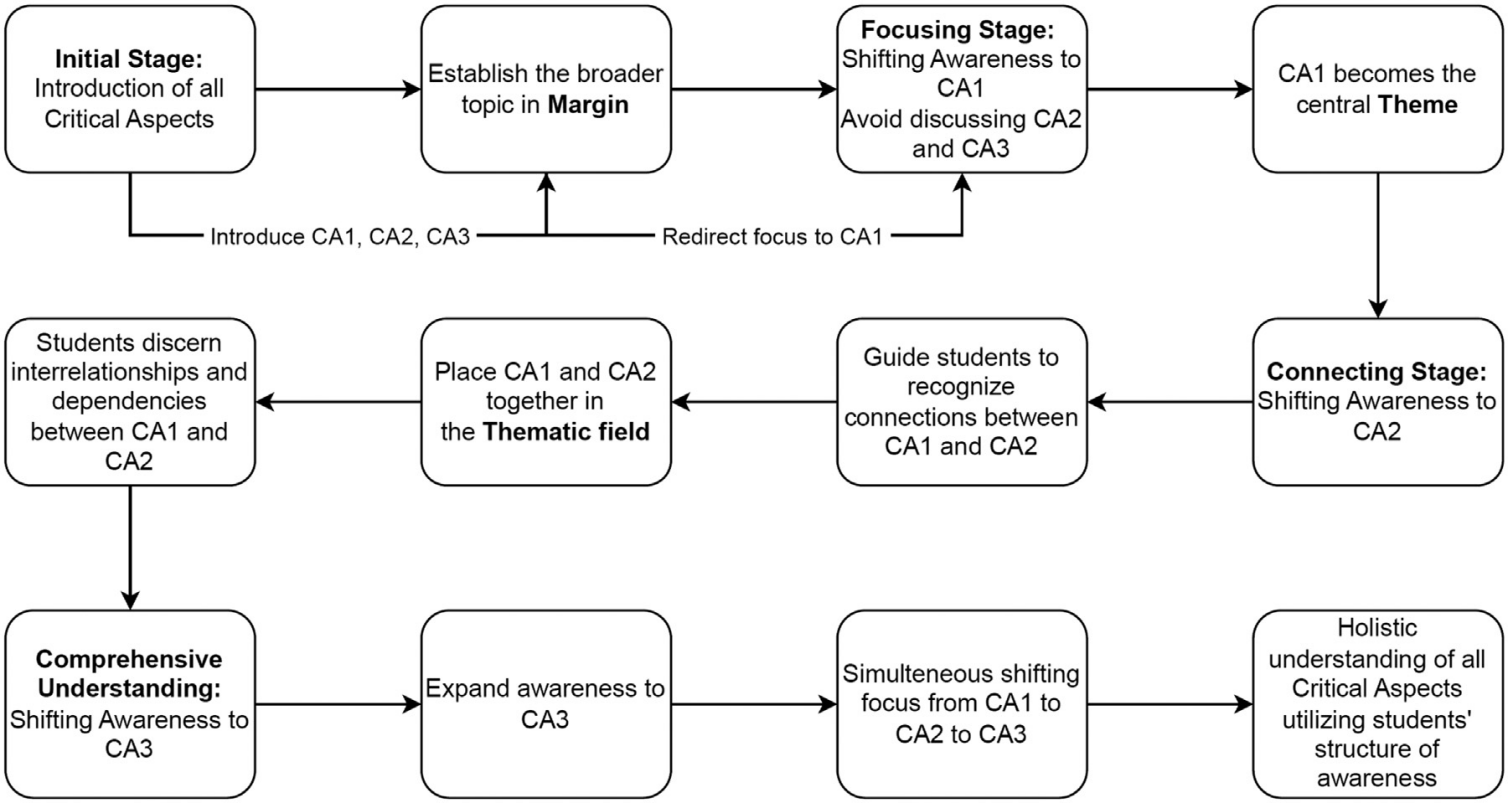
Implementation of the structure of awareness according to VTST method.

The procedure outlined above serves as an illustration of how instructors can intentionally manipulate students' awareness to draw attention to particular crucial elements and promote a deeper understanding of challenging ideas like photosynthesis. An explanation of how to implement/utilize students’ structure of awareness is presented in Fig. 5. During the first stage, all of the critical aspects (CA1, CA2, and CA3) are introduced within the Margin (See Figs. 4 and 5), so that the students are aware of them. This serves as the framework for their basic understanding of photosynthesis, the larger topic of discussion. The following stage entails the instructor drawing students' attention to a particular essential component, such as CA1 (e.g., “Sunlight as the Source of Energy”). By limiting discussion to CA1 and excluding CA2 and CA3, the teacher creates CA1 as the core theme or primary focus of students' awareness, which may be referred to as the Theme (See Figs. 4 and 5). This deliberate adjustment sharpens their concentration on the selected critical aspect. As learning proceeds, the teacher leads students to recognize the connections between CA1 and CA2, supporting the shift from CA1 to CA2 in their awareness, which may be referred to as Thematic Field. By contrasting these essential elements in the Thematic field, students come to recognize their interrelationships and interdependencies, thus enhancing their understanding. The following diagram illustrates a technique for teachers to integrate critical aspects and awareness structure within the framework of variation theory.

**Fig. 6:**
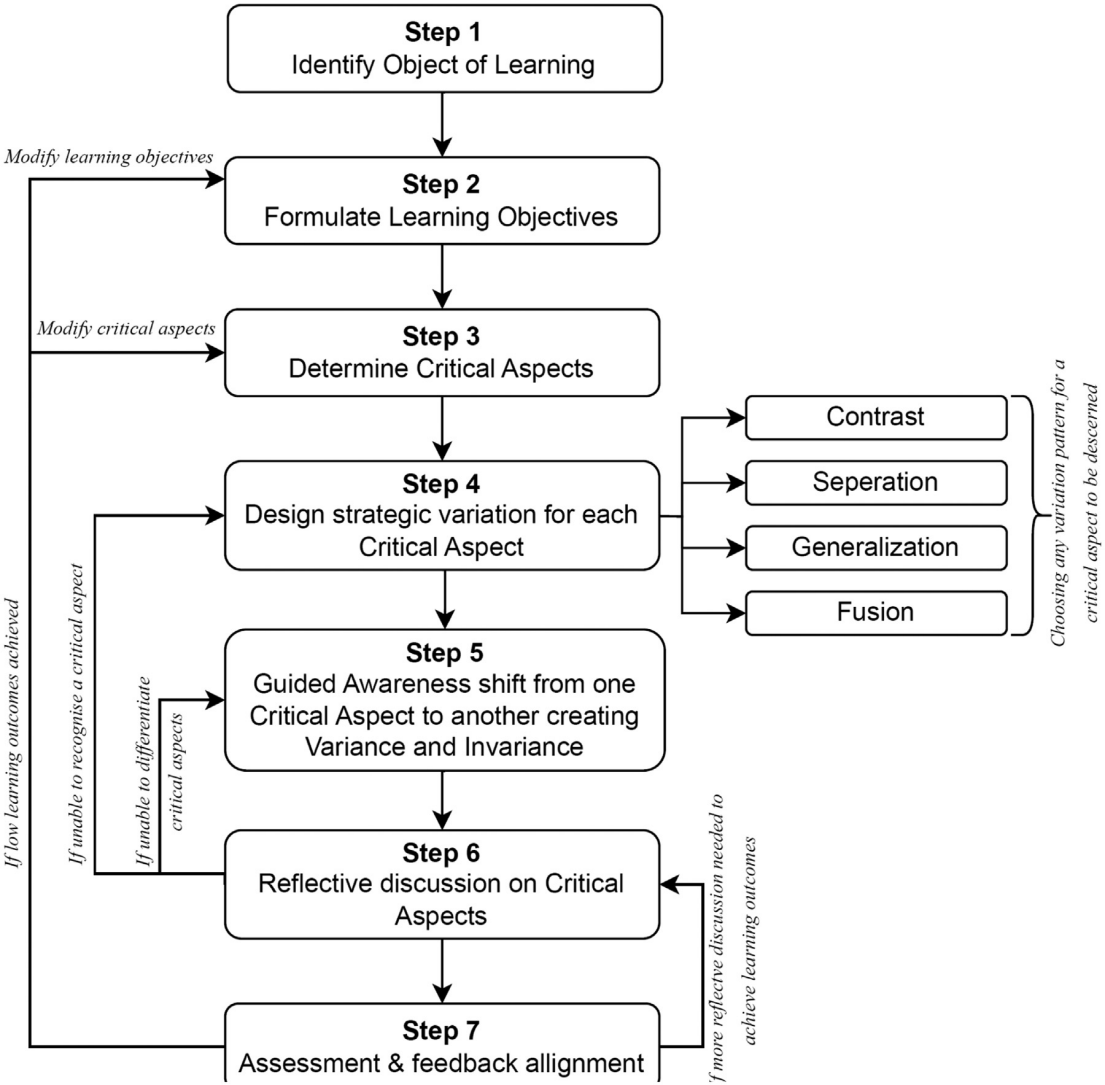
Essential steps to implement variation theory in STEM education.

After having an in-depth understanding of the key components of variation theory that have been discussed so far, teachers may endeavor to develop lesson plans for teaching STEM content. This article presents a comprehensive seven-step framework for developing a lesson plan following the VTST method (see Fig. 6). Initially, a flowchart diagram that describes the VTST method is presented in Fig. 6 followed by a general description of each step. Later, a practical guideline is presented directing how each step may be followed to develop a lesson plan for a STEM topic in general, and for an Engineering topic in particular for the reader of this article so the VTST methods may easily be replicated by other researchers in STEM education

### The seven-step model of the VTST method

*Step 1: Identify Object of Learning*

At the beginning, teachers need to identify the precise concept or subject matter within the realm of STEM education that serves as the primary focal point of the teaching process. The focal point of the learning experience will be centered on this item of study. The topic may encompass a scientific principle, mathematical notion, engineering technique, or any other subject within the scope of STEM.

*Step 2: Formulate Learning Objective*

Once the object of learning is identified, the teacher may focus on constructing clear and precise learning objectives that indicate what students should be able to comprehend, apply, and demonstrate by the end of the lesson or unit. These objectives must be directly aligned with the selected object of learning and serve as measurable outcomes that guide instruction and evaluation.

*Step 3: Determine Critical Aspects*

This stage is crucial for teachers to identify and present the critical aspects with their characteristics and dimensions in a sequential way that may help students understand well. Understanding the critical aspects are important element that helps students develop a complete understanding of the topic. They will act as key points for designing variations and directing students' attention.

*Step 4: Design Strategic Variation for Each Critical Aspect*

The four patterns of variation, namely contrast, separation, generalization, and fusion may be utilized, so students are able to discern the desired critical aspect. The lesson may begin with “Contrast” to stress key differences, “Separation” to expose interconnected effects, “Generalization” to draw out overarching principles, and “Fusion” to stress comprehensive comprehension. Utilizing the patterns of variations in lesson plans ensures the presentation of a critical aspect from different aspects.

*Step 5: Guided Awareness Shift from One Critical Aspect to Another*

Teachers may guide students' awareness from one critical aspect to another in a systematic manner. The lecture may begin by highlighting a certain important feature of a specific critical aspect. Then gradually move their focus to another critical aspect, providing opportunities for students to identify similarities and differences, promoting a more comprehensive understanding of the topic.

*Step 6: Reflective Discussion on Critical Aspects*

Opportunities for reflective conversations and activities are outlined in lesson plans. Teachers assist students in expressing their findings, trends, and conclusions drawn from the variances. Students are encouraged to actively process the concepts in relation to the critical aspect and improve their understanding throughout these discussions. Students may also be encouraged to provide recommendations on how to put newly learned skills to use in real-world circumstances, encouraging the transfer of knowledge. There are feedback loops between steps 6, 5, and 4 (Fig. 6). If the reflective discussion reflects a lack of understanding of a specific critical aspect, the teacher may go back to step 4. However, if students fail to express a holistic understanding of the topic, the teacher may go back to step 5.

*Step 7: Assessment and Feedback Alignment with Learning Objectives*

Create assessment tasks that are aligned with the learning objectives and the concepts of variation theory. Examine students' abilities to differentiate critical aspects, identify the patterns of variations, and apply their knowledge across several variants. Give students feedback that supports constructive thinking and helps them to refine their understanding. After the assessment, if students need a reflective discussion, the teacher may repeat step 6. However, if there are major concerns about the achievement of learning outcomes, teachers may go back to step 3 and modify the critical aspects, or step 2 and modify the learning objectives.

By following these steps, instructors in STEM education may systematically utilize variation theory to enhance deeper understanding among students. This systematic method ensures that students connect with critical aspects, recognize variations and invariances, and build a comprehensive grasp of complicated STEM topics.

### Developing lesson plan for an engineering topic using the seven-step VTST method

*Step 1: Identify Object of Learning*

The *object of learning* of this lesson is the Vapor Compression Refrigeration Cycle.

**Fig. 7:**
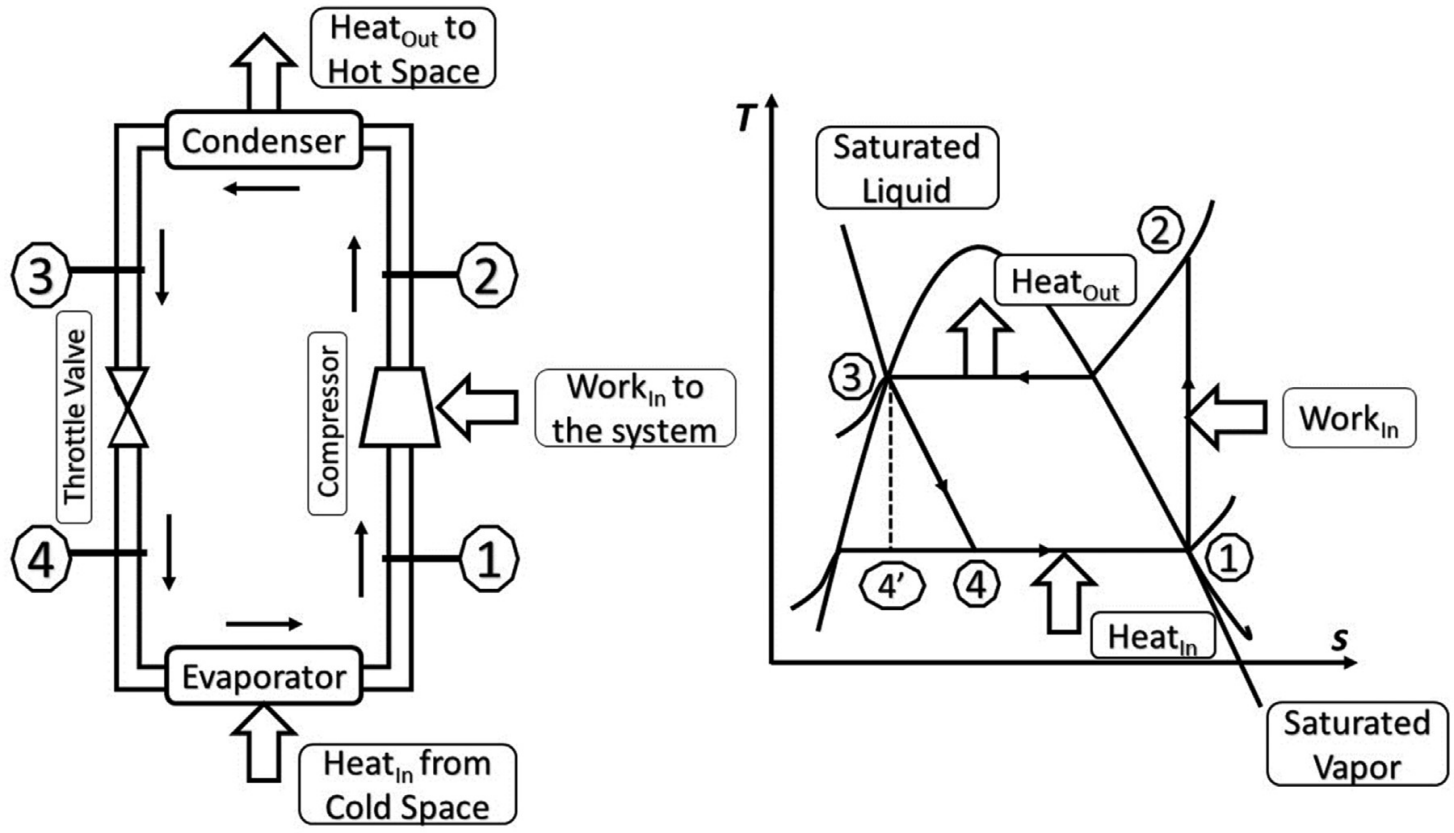
Schematic and T-s diagram for the ideal vapor-compression refrigeration cycle. *Adapted from*[25].

A schematic diagram of a simple Vapor Compression Refrigeration System along with a temperature–entropy diagram is presented in Fig. 7.

*Step 2: Formulate Learning Objectives*

### Trace refrigerant state changes in VCRS

At the end of the lesson, students will be able to identify the state changes of the refrigerant (vapor, liquid, and mixture) through the evaporator, compressor, condenser, and expansion valve in a vapor compression refrigeration System (VCRS).

*Step 3: Determine Critical Aspects*

(1). Critical Aspect 1: State of refrigerant in Compressor.
(2). Critical Aspect 2: State of refrigerant in Condenser.
(3). Critical Aspect 3: State of refrigerant in Expansion/Throttling Valve.
(4). Critical Aspect 4: State of refrigerant in Evaporator.

*Step 4: Design strategic variation for each Critical Aspect*

*Step 5: Guided Awareness shift from one Critical Aspect to another creating variance and invariance*

Steps 4 and 5 are the main steps where teachers utilize the patterns of variations and grab students’ awareness of the critical aspects one by one or simultaneously. The following section describes how steps 4 and 5 are implemented in STEM classrooms utilizing seven activities.

Activity 1: In this activity, the teacher may describe the critical aspect 1 (CA1), which is the compressor, including its basic functioning function, the different types of compressors, what occurs when vapor refrigerant enters the compressor, and the condition of the refrigerant after compression. The instructor also explains its application. The instructor may use lecture and discussion, and instructional materials may include PowerPoint presentation, multimedia projector, Whiteboard, and marker. At this stage, the state of the refrigerant before entering and after leaving the compressor will be contrasted while the discussion on the condenser, expansion valve, and evaporator will remain invariant. Table 1 represents the first activity of the lesson.

**Table 1:** Patterns of variation and invariance for refrigerant and compressor.

Activity	**CA1 Compressor**	CA2 Condenser	CA3 Expansion valve	CA4 Evaporator	State of Refrigerant	Patterns of variation and Invariance
1	**V**	I	I	I	V	The contrast in the refrigerant before entering and after leaving the compressor

*Note. V* = variation; *I* = invariance.

Activity 02: In this activity, the teacher may introduce critical aspect 2, which is the condenser, including its basic functions such as how the condenser exchanges temperature with the atmosphere and condenser cooling system, and the different varieties of condensers and their applications. The instructor also explains the state of the refrigerant through the condenser. The following questions also need to be highlighted by the teacher - How refrigerant is converted from vapor to liquid in the condenser? Which principles govern condensation? What does the term condensation mean? At this stage, the state of the refrigerant before entering and after leaving the condenser will be contrasted while the discussion on the compressor, expansion valve, and evaporator will remain invariant. Table 2 represents the second activity of the lesson.

**Table 2:** Patterns of variation and invariance for refrigerant and condenser.

Activity	CA1 Compressor	**CA2** **Condenser**	CA3 Expansion valve	CA4 Evaporator	State of Refrigerant	Patterns of Variation and Invariance
2	I	**V**	I	I	V	The contrast in the refrigerant before entering and after leaving the condenser

Activity 03: In this activity, the teacher may describe the expansion valve using an animation video and show different images of the expansion valve, for instance, the basic function of the expansion valve, how the expansion valve reduces pressure as the refrigerant flows through it, different types of expansion valves and their application. In addition, the instructor describes the state of the refrigerants in the expansion valve, how to shift high-pressure refrigerant to low-pressure refrigerant using various instances, and the principles that govern the expansion of refrigerant. At this stage, the state of the refrigerant before entering and after leaving the Expansion Valve will be contrasted while the discussion on the compressor, condenser, and evaporator will remain invariant. Table 3 represents the third activity of the lesson.

**Table 3:** Patterns of variation and invariance for refrigerant and expansion valve.

BB	CA1 Compressor	CA2 Condenser	**CA3** **Expansion valve**	CA4 Evaporator	State of Refrigerant	Patterns of Variation and Invariance
3	I	I	**V**	I	V	The contrast in the refrigerant before entering and after leaving the expansion valve

Activity 04: In this activity, the teacher will describe the evaporator using an animation video and show a different image of the evaporator, for example, the basic function of the evaporator, how the evaporator exchanges temperature with its surroundings, how to collect heat from the evaporator chamber, and how refrigerant evaporates in the evaporation, as well as the various types and applications of the evaporator. The instructor also describes how refrigerants evaporate in the evaporator and the process through which liquid refrigerant is transformed into vapor in the evaporator, which principles govern evaporation? And what does the term evaporation mean? At this stage, the state of the refrigerant before entering and after leaving the evaporator will be contrasted while the discussion on the compressor, condenser, and expansion valve will remain invariant. Table 4 represents the fourth activity of the lesson.

**Table 4:** Patterns of variation and invariance for refrigerant and evaporator.

Activity	CA1 Compressor	CA2 Condenser	CA3 Expansion valve	**CA4** **Evaporator**	State of Refrigerant	Patterns of Variation and Invariance
4	I	I	I	**V**	V	The contrast in the refrigerant before entering and after leaving the evaporator

In activities 1 to 4, among the four critical aspects, only one critical aspect was in the point of discussion retaining the remaining three as invariant. The teacher tries to grab students’ structure of awareness to one critical aspect at a time. To accomplish so, teachers usually employed contrast on the state of the refrigerant before and after passing through a component of the refrigeration system. In activities 5 and 6, the teacher can take more than one critical aspect at a time keeping the other two components constant. Here instructor can choose to build a separation between the compressor and expansion valve in activity 5, and also make a separation between the condenser and evaporation in activity 6. In both activities the functioning principle of the pair of components is opposite and therefore separation may be the preferred choice as the pattern of variation.

Activity 05: As discussed earlier, the working principle of the compressor and the expansion valve is just the opposite, where the refrigerant gains high pressure and temperature after passing through the compressor. Whereas the refrigerant loses its pressure and temperature after passing through the expansion valve. Utilizing the separation pattern of variation, the teacher would strategically isolate and emphasize the contrasting effects of the compressor and expansion valve on the refrigerant within the refrigeration cycle. Discussion of the compressor and expansion valve together, keeping the other two components invariant, allows students to discern the difference between the working principle of compressor and expansion valve. Table 5 represents the fifth activity of the lesson.

**Table 5:** Patterns of variation and invariance for refrigerant, compressor, and expansion valve.

Activity	**CA1** **Compressor**	CA2 Condenser	**CA3** **Expansion valve**	CA4 Evaporator	State of Refrigerant	Patterns of Variation and Invariance
5	**V**	I	**V**	I	V	The separation between the opposing working principles of compressor and expansion valve

Activity 06: Activity 6 is just a repetition of activity 5. In activity 6, instead of focusing on the compressor and the expansion valve, the other two components compressor and evaporator are presented together to the students’ structure of awareness. Before going into the condenser, the refrigerant has just left the compressor and is in the form of a high-pressure vapor. As the refrigerant moves through the condenser, a big change happens: the refrigerant loses heat to the environment, which causes it to change. At this point, when the refrigerant goes through the condenser, it changes from a high-pressure gas to a high-pressure liquid. By switching the focus to the evaporator, students can see how the evaporator works differently from the condenser. Before going into the evaporator, the refrigerant is in the form of a high-pressure liquid. This is because the condenser has cooled it down. As the refrigerant moves through the evaporator, it picks up heat from its surroundings. In this case, the refrigerant goes from being a liquid under high pressure to a gas under low pressure. Table 6 represents the sixth activity of the lesson.

**Table 6:** Patterns of variation and invariance for refrigerant, condenser, and Evaporator.

Activity	CA1 Compressor	**CA2** **Condenser**	CA3 Expansion valve	**CA4** **Evaporator**	State of Refrigerant	Patterns of Variation and Invariance
6	I	**V**	I	**V**	V	The separation between the opposing working principles of condenser and Evaporator

Activity 07: Through Activity 01 to 04 students got familiar with the working principle of each critical aspect separately by contrasting the state of the refrigerant before and after each component. Hence students got a nuanced grasp of the working principle of each component. Afterwards, in activities 05 to 06 students were able to discern the separation between the working principles of two sets of components. Now, at this stage, using a fusion pattern of variation, the teacher tries to gather all concepts together to make a holistic understanding of the VCRS, illustrating the focus of the state change before and after all four components. The compressor drives the refrigerant from a low-pressure to a high-pressure vapor, giving it the energy, it needs for the following steps. A crucial transition from a high-pressure vapor to a high-pressure liquid, which releases heat into the surroundings, takes place as the refrigerant moves toward the condenser. The refrigerant undergoes this transformation to get ready for the expansion valve, where it changes from a high-pressure liquid to a low-pressure vapor. As the refrigerant absorbs heat to complete the cycle, the evaporator converts it from low-pressure vapor into a low-pressure liquid.

At the end of Activity 07, it is expected that students understand how in the Vapor Compression Refrigeration System, the compressor, condenser, expansion valve, and evaporator function as interlocking puzzle pieces to create a fascinating journey by changing their state as they pass through each component. Table 7 represents the seventh activity of the lesson.

**Table 7:** Patterns of variation and invariance for refrigerant, and all critical aspects.

Activity	**CA1** **Compressor**	**CA2** **Condenser**	**CA3** **Expansion valve**	**CA4** **Evaporator**	**State of Refrigerant**	Patterns of Variation and Invariance
7	**V**	**V**	**V**	**V**	**V**	The Fusion of all components as variants with the state of refrigerant

Table 8 shows a summary of the seven activities as a part of the lesson planning steps 4 and 5 according to VTST method. Students' learning experiences are extended and deepened when the Vapor Compression Refrigeration Cycle (VCRS) is taught using the principles of variation and invariance. Students are encouraged to actively engage with the content by gradually introducing variations and invariance in how each element of the cycle operates and affects the refrigerant. These modifications encourage students to examine and distinguish the various roles and outputs of the compressor, condenser, expansion valve, and evaporator.

**Table 8:** Summary of Activities of a lesson plan using VTST method.

Activity	CA1 Compressor	CA2 Condenser	CA3 Expansion valve	CA4 Evaporator	State of Refrigerant	Patterns of Variation and Invariance
1	**V**	I	I	I	V	The contrast in the refrigerant before entering and after leaving the compressor
2	I	**V**	I	I	V	The contrast in the refrigerant before entering and after leaving the condenser
3	I	I	**V**	I	V	The contrast in the refrigerant before entering and after leaving the expansion valve
4	I	I	I	**V**	V	The contrast in the refrigerant before entering and after leaving the evaporator
5	**V**	I	**V**	I	V	The separation between the opposing working principles of compressor and expansion valve
6	I	**V**	I	**V**	V	The separation between the opposing working principles of condenser and Evaporator
7	**V**	**V**	**V**	**V**	**V**	The Fusion of all components as variant with the state of refrigerant

*Step 6: Reflective discussion on Critical Aspects*

Referring to Fig. 6, after completing step 5, the teacher encourages a reflective conversation on the critical aspects by helping students recall the diverse perspectives provided during the session. Reflective conversation develops critical thinking and enhances students' capacity to apply their enriched understanding to real-world settings, building a comprehensive grasp of the concept's complexity. The prime objective of this discussion is to check if students were able to discern the individual critical aspects, and whether they were able to differentiate among the critical aspects, which is the changing state of the refrigerant through the components of the VCRS, in this case. Refer to Fig. 6, if students are unable to discern the working principle of the compressor, condenser, expansion valve, or evaporator, the teacher may review step 4, and if students can recognize a critical aspect individually, but are unable to differentiate among them, then the teacher may focus on step 5.

*Step 7: Assessment and feedback alignment*

Referring to Fig. 6, the final step in implementing variation theory is to develop assessment and feedback that align with the learning objectives formulated in step 2. Carefully crafting questions considering the domain of learning is the key to developing tests, which can be used as an instrument to assess students' learning outcomes. If the teacher thinks that more reflective discussion is needed to make students’ concepts clear, they may follow step 6. However, if the assessment reflects low learning outcomes, then the teacher may need to modify the critical aspects (step 3) or even the learning objectives (step 2).

Overall, the interaction of variation and invariance in teaching the VCRS promotes a thorough comprehension of the complex mechanics of the cycle. Students are given the skills necessary to recognize underlying concepts and comprehend how various elements work together holistically. This method not only encourages active learning but also helps students develop the critical thinking abilities needed to apply their knowledge in real-world situations, improving their capability to identify and resolve refrigeration system problems.

## Method validation

Despite different methods exist, however, when considering the evaluation of achieved learning outcomes, particularly in settings that involve the utilization of variation theory, it is recommended to employ a specific assessment approach known as phenomenography. This method was initially developed by Ference Marton and his research team at the University of Gothenburg in Sweden [26]. In accordance with the suggested phenomenographic approach, the authors of this scholarly publication, in collaboration with three more researchers, conducted an extensive investigation. The research utilized variation theory in the field of engineering, and later assessed the learning outcomes using the phenomenographic framework. The research findings were presented at the conference conducted by the European Society for Engineering Education (SEFI, 2022) held in Barcelona, Spain [27]. The inclusion of this paper in a highly regarded international academic forum serves as a recognition and affirmation of the validity of the method by a wide and influential academic community in the European region. The following table provides a concise summary of the research outcomes, effectively summarizing the core findings of the study.

Two groups of students were the sample of this study. Using a traditional method, an instructor presented the material to a control group, followed by a 30-minute student response window. Also, using variation theory, another instructor presented an identical subject to an experimental group. Both groups were given the same written question for evaluation, with the goal of determining the impact of variation theory on their understanding of the learning objective. Table 9 depicts the differences in how the experimental and control groups understood the object of learning (which was “production line quality control” in this case), as influenced by the use of variation theory or not. The majority of the experimental cohort correctly identified all four characteristics taught by variation theory, but the control group struggled with higher-order concepts. This study validates the variation theory as a method of teaching by confirming that it efficiently cultivates favorable learning environments, resulting in a positive impact on engineering education by improving students' understanding.

**Table 9:** Comparison of learning outcome achieved in presence and absence of variation theory.

		*Experimental Group (37 students) (Vanation theory applied)*		*Control Group (26 students) (Variation theory NOT applied)*	
Conception		Occurrences	%	Occurrences	%
Lower-order conceptions	A	34	91.9	22	84.6
	B	30	81.1	16	61.5
Higher-order conceptions	C	34	91.9	1	3.9
	D	36	97.3	1	3.9

Adapted from the study of M. Hasan et al. [27].

## Conclusion

This article reviews the key components of the variation theory and provides a guideline about its implementation. Particularly it discusses how the complex STEM topics could be taught in such a systematic way so that the STEM teachers could replicate the proposed VTST method. From the learning perspective, this article creates a profound basis for an argument that if teachers can develop lesson plans utilizing the VTST method, it will create the necessary conditions for learning and help students comprehend the complex problems of STEM education. Researchers in the field of STEM education can take this article as a reference to develop authentic learning experiences for students. Future research may utilize the sample lesson plan presented in this article and further investigate the effectiveness of VTST adapting a phenomenographic approach. Another future research avenue could be the integration of other methods with VTST to investigate their usefulness for more favorable student learning.

## CRediT authorship contribution statement

**Mahbub Hasan:** Conceptualization, Methodology, Writing – original draft, Writing – review & editing, Project administration. **Md Shahadat Hossain Khan:** Conceptualization, Methodology, Writing – review & editing. **A.K.M. Foysal Ahmed:** Conceptualization, Methodology, Writing – review & editing.

## Declaration of Competing Interest

The authors declare that they have no known competing financial interests or personal relationships that could have appeared to influence the work reported in this paper.

## Data Availability

Data will be made available on request.
